# Calcium signaling in crops

**DOI:** 10.1111/nph.70796

**Published:** 2025-12-09

**Authors:** Chunxia Zhang, Yang Song, Jörg Kudla

**Affiliations:** ^1^ Key Laboratory of Plant Molecular Physiology Institute of Botany, Chinese Academy of Sciences Beijing 100093 China; ^2^ China National Botanical Garden Beijing 100093 China; ^3^ College of Agronomy Jilin Agricultural University Changchun 130118 China; ^4^ Institut für Biologie und Biotechnologie der Pflanzen Universität Münster Münster 48149 Germany; ^5^ National Key Laboratory of Crop Genetic Improvement Huazhong Agricultural University Wuhan 430070 China

**Keywords:** calcium signaling, crop improvement, gene editing, smart crops, stress resilience, synthetic genetic variation

## Abstract

Calcium (Ca^2+^) signaling is integral to nearly all aspects of plant biology, including development and responses to biotic and abiotic stresses. It operates through two main layers: the generation of Ca^2+^ signals and their decoding by Ca^2+^‐binding proteins, which act early in diverse signaling pathways. The system exhibits remarkable robustness and versatility, largely due to its network‐like organization. While fundamental principles of Ca^2+^ signaling were initially established in noncrop model organisms, recent research has increasingly expanded toward major crop species and has demonstrated that natural and synthetically created variation in Ca^2+^ signaling components can shape agronomically important traits. In this review, we first provide a concise overview of the fundamental principles of plant Ca^2+^ signaling and then synthesize the current status of this research field in major crop plants. We discuss why exploiting existing natural and engineering synthetic genetic diversity in Ca^2+^ signaling components offers promising strategies to enhance crop stress resilience and yield stability. Subsequently, we delineate how – aided by artificial intelligence – superior alleles can be identified and/or created and incorporated into elite crop genomes. Finally, we discuss current challenges and emerging perspectives in translating Ca^2+^ signaling research into practical applications for crop improvement.

**Table:** 

	Contents	
	Summary	1644
I.	Introduction	1644
II.	The current status of plant Ca^2+^ signaling research – implications from studying model species and their relevance for crops	1645
III.	A conceptual roadmap for harnessing the potential of Ca^2+^ signaling for crop improvement	1650
IV.	Current challenges and future perspectives for Ca^2+^ signaling research in crops	1654
	Acknowledgements	1655
	References	1655

## I. Introduction

Calcium (Ca^2+^) serves as a universal and versatile second messenger in eukaryotes (Clapham, 2007). The concerted action of Ca^2+^ channels conferring Ca^2+^ influx, resulting in a transient elevation of cytosolic Ca^2+^ concentration and Ca^2+^ transporters mediating reverse Ca^2+^ efflux from the cytosol, creates spatio‐temporally defined Ca^2+^ signals (Sanders *et al*., 2002; Dodd *et al*., 2010; Kudla *et al*., 2010). Ca^2+^ influx across the plasma membrane occurs primarily via Ca^2+^‐permeable channels, including cyclic nucleotide‐gated channels (CNGCs), glutamate receptors (GLRs), and mechanosensitive channels (MSLs, MCAs, and OSCAs) (Kudla *et al*., 2018; Brownlee & Wheeler, 2025). Nucleotide‐binding leucine‐rich repeat receptors (NLRs) mediate immune responses, including cell death, and recent work has revealed that plant NLRs form Ca^2+^ pores involved in Ca^2+^‐mediated resistance to pathogen attack (Lee & Romeis, 2022). In the legumes *Medicago trunculata* and *Lotus japonicus*, Nod factor perception by LysM‐type plasma membrane receptors causes ER‐derived nuclear Ca^2+^ elevation. In this case, interaction between CNGC15 and the CASTOR/POLLUX/DMI1 Ca^2+^‐permeable channels on the inner nuclear envelope/membrane underlies the release of Ca^2+^ into the nucleoplasm (Charpentier, 2018; Brownlee & Wheeler, 2025). Two main classes of Ca^2+^‐ATPases remove Ca^2+^ from the cytosol to restore resting Ca^2+^ concentrations. The first class, Type IIA ATPases (ER‐type, ECA), are mainly located on endomembranes. The second class, Type IIB autoinhibited ATPases (ACA), can be located on both the plasma membrane (PM) and endomembranes, including the vacuole, endoplasmic reticulum (ER), and Golgi apparatus (Kudla *et al*., 2018; Costa *et al*., 2023; Brownlee & Wheeler, 2025).

Plants possess an extensive array of Ca^2+^‐binding proteins that decode and transmit these signals. These plant Ca^2+^ sensor proteins include calmodulins (CaM), calmodulin‐like proteins (CMLs), Ca^2+^‐dependent protein kinases (CDPKs or CPKs), calcineurin B‐like proteins (CBLs) and their interacting kinases (CIPKs), as well as Ca^2+^/calmodulin‐dependent kinases (CCaMKs) (Kudla *et al*., 2018). Additionally, Ca^2+^ directly regulates the activity of other proteins, such as respiratory burst oxidase homolog proteins (RBOHs) and the vacuolar two‐pore channel TPC1 (Hedrich *et al*., 2023; X. Zhang *et al*., 2025). Generally, Ca^2+^ signals function very early and rather upstream in response and adaptation processes and are involved in almost all aspects of plant physiology, development, and their responses to environmental cues (Sanders *et al*., 2002; Kudla *et al*., 2018; Wang & Luan, 2024). It governs plant development, already beginning with pollen tube growth and during fertilization, controls cell and tissue differentiation, and regulates general plant growth but also the growth and differentiation of specialized cells such as root hairs (Konrad *et al*., 2011; Denninger *et al*., 2014; Kudla *et al*., 2018; T. Li *et al*., 2019; Tian *et al*., 2020). Historically, early discoveries and reports on Ca^2+^ signals in plants directly linked this phenomenon to agronomically important processes, such as cold stress responses, stomatal regulation in drought tolerance, symbiotic interactions, and defense responses conferring plant immunity (McAinsh *et al*., 1990; Knight *et al*., 1991; Nürnberger *et al*., 1994; Ehrhardt *et al*., 1996; Allen *et al*., 2001; Navazio & Mariani, 2008). The concurrent role of Ca^2+^ signaling in responses to biotic and abiotic environmental stimuli makes this research area particularly important for improving our understanding of how plants cope with multiple stresses simultaneously.

The subsequent exploration of components and mechanisms underlying the formation and decoding of Ca^2+^ signals advanced rapidly in suitable model systems, including Arabidopsis and nitrogen‐fixing legumes. In recent years, however, this research has expanded to a broader range of crop plants. A comparison of Ca^2+^ signaling in model species and crops indicates considerable conservation and commonalities of the involved components (Edel & Kudla, 2015). However, it also reveals notable differences and deviations regarding their regulation and function in different species. Crop yield is largely determined by a plant's ability to withstand abiotic and biotic stresses and to optimally adapt to available resources and environmental conditions. The upstream, transient, and ubiquitous function of Ca^2+^ signaling in these processes warrants consideration of this facet of crop biology as an important subject of agronomically relevant research and as a potential target of crop improvement.

In this review, we initially reflect on recent key insights on Ca^2+^ signaling and summarize the current status of this research in various crop species. To aid in orientation, we use gene/protein names without a species indication (e.g. CBL4) when referring to Arabidopsis, but we include the species abbreviation (e.g. OsCBL4 for rice CBL4) when referring to another species. For the benefit of clarity, we focus on selected illustrative examples, such as salt stress responses and guard cell regulation, as well as pathogenic and symbiotic interactions of plants, as model cases, instead of making an attempt at comprehensiveness. Building on this, we contemplate how and why harnessing the naturally existing genetic variation and engineering synthetic genetic variation of Ca^2+^ signaling components in crops could offer novel opportunities to optimize stress resilience and yield stability. We also propose experimental strategies along these lines and discuss their potential to create smart crops that can autonomously mount specific and optimal responses to environmental changes. Finally, we will attempt to deduce the resulting challenges and prospects for future research on Ca^2+^ signaling in crops.

## II. The current status of plant Ca^2+^ signaling research – implications from studying model species and their relevance for crops

**Fig. 1:**
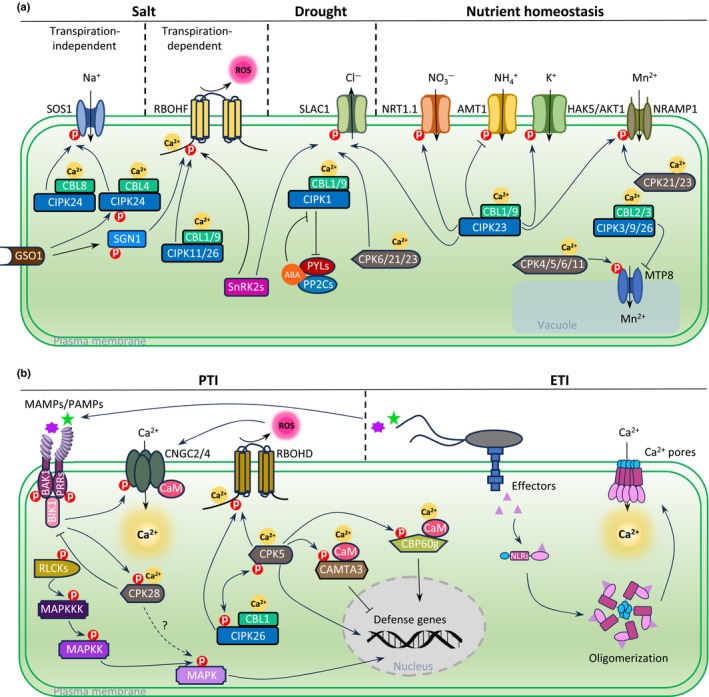
Functions of Ca^2+^ signaling in stress responses in Arabidopsis. (a) Schematic illustration of Ca^2+^ signaling involved in responses to salt, drought stresses, and nutrient homeostasis. (b) Ca^2+^ signaling in pathogen‐associated molecular pattern (PAMP)‐triggered immunity (PTI) and effector‐triggered immunity (ETI). Further details are provided in the main text.

Recent comprehensive reviews have detailed the current state of plant Ca^2+^ signaling research (Edel *et al*., 2017; Luan & Wang, 2021; Köster *et al*., 2022; Lee & Romeis, 2022; Costa *et al*., 2023; Brownlee & Wheeler, 2025). Key principles of plant Ca^2+^ signaling, which are particularly relevant to the topics discussed in this review, are summarized in Fig. 1 and can be generalized in a simplified way as follows. (1) Multiple Ca^2+^ signaling mechanisms can function in one specific response pathway. (2) Plant Ca^2+^ signaling systems exhibit network‐like characters including flexibility and robustness. (3) In contrast to the situation in animals, formation of a stimulus‐specific plant Ca^2+^ signal appears not to be dominated by a few prominently functioning Ca^2+^ channel types, but instead appears to involve the contribution of several Ca^2+^ channel family members or even multiple Ca^2+^ channel types. (4) Ca^2+^ signals are decoded by few families of Ca^2+^ sensors and Ca^2+^‐regulated kinases, which can have many members. (5) Members of the Ca^2+^ sensor and Ca^2+^‐regulated kinase families can exhibit different Ca^2+^ binding affinities or dissociation constants, which enables the differential decoding of Ca^2+^ signals. (6) One Ca^2+^‐regulated kinase can regulate multiple targets, and multiple Ca^2+^‐regulated kinases can converge on one target. (7) Ca^2+^‐regulated kinases can work in concert with Ca^2+^‐independent kinases, can be regulated by other kinases, and can, in turn, regulate other kinases. (8) The role and regulation of phosphatases counteracting kinases in Ca^2+^ signaling remain underexplored. Most of these principles were initially derived from research on model species (DeFalco *et al*., 2009; Kudla *et al*., 2018; Dong *et al*., 2022; Lederer *et al*., 2025). However, as discussed below, accumulating evidence suggests that a similar framework of components and mechanisms governs Ca^2+^ signaling processes in crops.

### 1. Ca^2+^ signaling in response to abiotic environmental cues

One well‐studied function of Ca^2+^ signaling is its role in plant tolerance to salt (NaCl). Studies on tobacco and Arabidopsis have shown that exposure to elevated concentrations of NaCl triggers rapid, transient primary Ca^2+^ signals in specific regions of the root that subsequently convert into wave‐like systemically propagating Ca^2+^ signals (Choi *et al*., 2014; Steinhorst *et al*., 2022). These Ca^2+^ signals are thought to activate the ‘salt overly sensitive’ (SOS) pathway for sodium detoxification. In this pathway, the Ca^2+^ sensor SOS3/CBL4 interacts with SOS2/CIPK24 to activate the Na^+^ : H^+^ antiporter SOS1 at the plasma membrane, which extrudes Na^+^ from the cell, thus enhancing salt tolerance (Fig. 1a) (Yang & Guo, 2018). This core pathway for salt tolerance is conserved in crops such as tomato, maize, and rice (Köster *et al*., 2019). Accordingly, homologous rice genes were shown to complement Arabidopsis *sos* mutants, and maize *cipk24* mutants displayed salt‐sensitive phenotypes (Köster *et al*., 2019; Zhou *et al*., 2022). However, notable differences also exist in Ca^2+^‐controlled salt stress tolerance mechanisms between Arabidopsis and major crops. In *Arabidopsis*, elevated salt stress triggers enhanced Ca^2+^ signals, which, beyond a specific threshold, activate the Ca^2+^ sensor CBL8. This activation occurs in addition to the baseline activation of CBL4 under moderate salt stress. Thus, in *Arabidopsis* roots, the SOS3/CBL4–SOS2/CIPK24–SOS1 axis confers basal salt tolerance, while the CBL8–SOS2/CIPK24–SOS1 module is activated only under severe salt stress (Fig. 1a) (Steinhorst *et al*., 2022). Notably, CBL8 orthologs are absent in monocots (including key cereal crops like rice, maize, and wheat), suggesting that this Ca^2+^‐sensor switch mechanism for elevated salt stress tolerance does not naturally occur in cereals. Therefore, the ubiquitous expression of CBL8 from dicots in cereals could offer a promising strategy to enhance salt tolerance in these crops.

Recent studies in *Arabidopsis* have further elucidated the regulation of CIPK24 and its integration with other signaling pathways, particularly in coordinating salt detoxification with stress‐induced developmental plasticity. The activity of the CBL4/SOS3–SOS2/CIPK24 complex is not solely regulated by Ca^2+^ signals but also by interactions with other proteins, such as GIGANTEA, which enables coordination between salt tolerance and flowering time regulation. Furthermore, phosphorylation of PLETHORA1/2 by SOS2 protects primary root growth and meristem maintenance during salt stress (Yang & Guo, 2018; Hao *et al*., 2023). Notably, phosphorylation/dephosphorylation of SOS2 by other kinases such as GSO1 and GRIK1, as well as additional unknown kinases and phosphatases, emerges as a key mechanism for tuning SOS2 kinase activity in specific cell types or response situations and for integrating SOS2 function with other signaling pathways (Köster *et al*., 2019; Chen *et al*., 2023). For example, the GSO1/SGN3‐SGN1‐RBOHF axis regulates root endodermal lignification, which facilitates transpiration‐dependent salt tolerance, while the GSO1‐SOS2‐SOS1 axis enhances Na^+^ extrusion from the meristem (Fig. 1a) (Fujita *et al*., 2020; Chen *et al*., 2023). Additionally, phosphorylation of SOS2 at S294 by an unknown kinase maintains its reduced activity, enabling interaction with specific 14‐3‐3 proteins, which are regulated by Ca^2+^ binding and degraded upon salt stress exposure (Köster *et al*., 2019). Understanding whether similar mechanisms of SOS2 activity regulation exist in crops represents an important area for future research that clearly deserves to be investigated. Since DNA triplets encoding phosphorylation sites are principally amenable to site‐directed mutagenesis through precision‐editing or homology‐directed repair (HDR)‐mediated editing, such regulatory sites may serve as targets for synthetic crop optimization in the future.

**Fig. 2:**
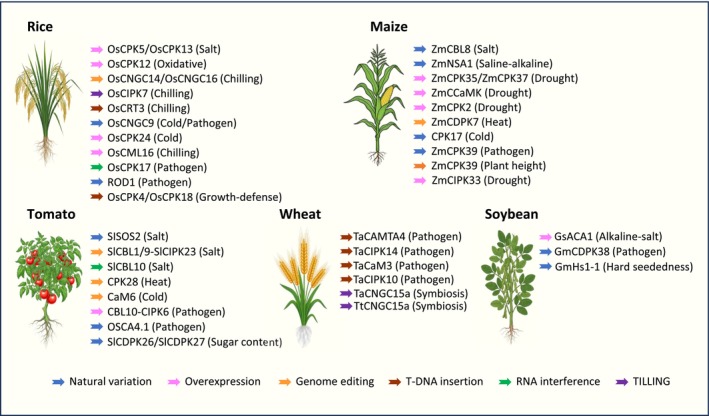
Experimentally characterized potential target genes for developing stress‐resilient crops. Illustration depicting Ca^2+^ signaling‐related genes that have been characterized as being associated with abiotic stress responses, pathogen defense, and growth‐related traits in major crops: rice (*Oryza sativa*), maize (*Zea mays*), soybean (*Glycine max*), tomato (*Solanum lycopersicum*), and wheat (*Triticum aestivum*/*Triticum turgidum*). The color code of the arrows is indicated at the bottom of the figure and indicates the experimental approach that was used to characterize gene function. Further details and references are provided in Supporting Information Table S1.

Recent studies provided the first evidence for the existence of physiologically relevant natural variation affecting the function of the SOS pathway in certain crops (Fig. 2; Supporting Information Table S1). In tomato, natural variation in the *SlSOS2* promoter region has been linked to changes in the root Na^+^ : K^+^ ratio and the loss of salt resistance during domestication. This variation affects an ABI4‐binding *cis*‐element that regulates the expression of *SlSOS2* (Hong *et al*., 2023). Additionally, a variant in the promoter of *SlSOS1*, which was selected during domestication and correlates with reduced salt tolerance, was recently identified. This variation disrupts a SlDREB2‐binding *cis*‐element, leading to decreased expression of SlSOS1 (Z. Wang *et al*., 2021). The SOS pathway has also been shown to play a conserved role in salt tolerance in maize. Specifically, natural genetic variation in two components of the SOS pathway, ZmSOS1 and ZmCBL8 (a homolog of *Arabidopsis* CBL4), is involved in the regulation of Na^+^ homeostasis and salt tolerance. In the case of ZmCBL8, an LTR/Gypsy promoter insertion reduces its expression, thereby increasing shoot Na^+^ concentration (Zhou *et al*., 2022). These findings provide examples illustrating the potential for enhancing stress tolerance in crops via genome editing of promoter *cis*‐regulatory elements (CREs) for tuning gene expression of Ca^2+^ signaling genes.

A recent study involving a chromosome‐scale genome assembly of *Hordeum brevisubulatum* and resequencing of 38 related crop wild relative (CWR) accessions underscores that expanding the exploration of natural variation in Ca^2+^ signaling beyond domesticated crop varieties to include CWRs has immense potential for crop improvement (Feng *et al*., 2025). Among Triticeae CWRs, the *Hordeum*‐I genome species *H. brevisubulatum* has attracted particular attention due to its exceptional stress tolerance, particularly to saline and alkaline‐saline soils. Genomic analyses, combined with functional studies, revealed that stress‐adaptive evolution in *H. brevisubulatum* involved the duplication of a Ca^2+^‐responsive stress sensor–responder CaBP‐NRT2 module. Notably, the previously uncharacterized EF‐hand‐containing Ca^2+^‐binding protein (CaBP) is present in two copies in the genome of salt‐sensitive *H. vulgare*, but expanded to eight copies in the genome of *H. brevisubulatum*. Importantly, overexpressing one of these CaBP genes from *H. brevisubulatum* in *H. vulgare* significantly increased its alkaline salt tolerance and enhanced grain yield by 28.7%, suggesting that CaBP confers salt tolerance through increased gene expression dosage (Feng *et al*., 2025). These findings highlight the potential for enhancing alkaline stress tolerance in other crops through enhanced CaBP expression, which could be achieved via genome editing of *cis*‐regulatory elements (CREs) in promoters of CaBP genes to boost their gene expression. Additionally, in the genome of *H*. *brevisubulatum*, the locus encoding the CBL/CIPK‐regulated SOS1 Na^+^ : H^+^‐antiporter, a well‐established determinant of salt tolerance, expanded to five copies compared with just one in *H. vulgare*. However, the physiological significance of this gene duplication for salt tolerance remains to be experimentally validated (Feng *et al*., 2025). Collectively, these studies not only establish the existence of valuable natural genetic resources for breeding salt‐resistant crops, but also demonstrate that Ca^2+^ signaling components offer important targets for breeding and for synthetically creating salt stress‐tolerant crops.

The potential to leverage natural variation in Ca^2+^ signaling genes is certainly not limited to improving salt tolerance. A groundbreaking study identified a single‐nucleotide mutation in the *CHILLING TOLERANCE DIVERGENCE 1* (COLD1gene), which originated from Chinese wild populations of *O. rufipogon*, as conferring adaptation of japonica rice to chilling. Further molecular characterization revealed that COLD1 localizes to the plasma membrane and endoplasmic reticulum (ER), where it plays a role in sensing cold and triggering Ca^2+^ signals for chilling tolerance (Ma *et al*., 2015). Additional research on cold tolerance in rice highlighted the multifaceted mechanisms by which Ca^2+^ signaling components impact stress tolerance (Fig. 2). The ER‐resident Ca^2+^‐regulated chaperone *Calreticulin 3* (OsCRT3) functions as a positive regulator of cold tolerance, mediating increases in cytosolic Ca^2+^ levels and interaction‐dependent activation of OsCIPK7 under cold stress (Guo *et al*., 2023). Moreover, an independent TILLING approach identified a single‐nucleotide mutation in the activation domain of OsCIPK7, which enhanced kinase activity and improved rice cold tolerance, offering a potential target for precision‐editing strategies in various crops (Zhang *et al*., 2019). Another study found that overexpression of the CDPK *OsCPK24* increased rice cold tolerance by promoting antioxidant accumulation (Liu *et al*., 2018).

While some Ca^2+^‐regulated kinases, such as CIPK24, appear to play a prominent role in one specific stress response pathway, accumulating evidence suggests that many others contribute to multiple stress responses. One such example is CPK5, a CDPK in *Arabidopsis* that regulates various targets across different processes. CPK5, for instance, regulates the vacuolar manganese transporter MTP8, but during pathogen infection, it activates the NADPH oxidase RBOHD to promote ROS production and also mediates transcriptional regulation by phosphorylating CAMTA3 and CBP60g (Fig. 1a,b) (Dubiella *et al*., 2013; Ju *et al*., 2022; Liu *et al*., 2024). Similarly, CIPKs also demonstrate versatility in substrate regulation. Among the CIPKs, CIPK23 stands out for its involvement in regulating a wide range of ion transporters and channels to control and coordinate plant nutrition in Arabidopsis. Additionally, CIPK23 regulates guard cell transpiration by phosphorylating the ion channel SLAC1 (Fig. 1a) (Maierhofer *et al*., 2014; Hedrich & Geiger, 2017; Manishankar *et al*., 2018; Dong *et al*., 2021). Furthermore, the regulation of MTP8 by at least four CPKs and three CIPKs underscores the intricate nature of Ca^2+^ signaling, highlighting its complexity and interdependence (Fig. 1a) (Zhang *et al*., 2021; Ju *et al*., 2022). Collectively, these examples illustrate the versatile and multifaceted role of Ca^2+^ signaling in plants. While this complexity offers exciting possibilities for crop improvement, it also presents significant challenges that must be carefully considered in efforts to harness Ca^2+^ signaling for agricultural applications.

### 2. Ca^2+^ signaling in plant immunity and symbiosis

A central and multifaceted role of Ca^2+^ signaling is also well documented in plant immunity. In the first layer of the plant immune system, cell surface‐localized pattern recognition receptors (PRRs) perceive microbe/pathogen‐associated molecular patterns (MAMPs/PAMPs) or damage‐associated molecular patterns (DAMPs) to initiate pattern‐triggered immunity (PTI) (Jones & Dangl, 2006). In Arabidopsis, Ca^2+^ signaling impacts PTI in several ways, including the elevation of cytosolic Ca^2+^ concentration, the promotion of reactive oxygen species (ROS) production through RBOHD activation by CBL1/CIPK26 and CPK5, crosstalk with mitogen‐activated protein kinase (MAPK) cascades, and transcriptional reprogramming (Fig. 1b) (Köster *et al*., 2022; Wang & Luan, 2024). Specifically, CBL1/CIPK26 and CPK5 work in concert with the receptor‐like cytoplasmic kinase (RLCK) BIK1 to phosphorylate RBOHD, facilitating ROS‐mediated systemic immunity (Dubiella *et al*., 2013; Kadota *et al*., 2014; Li *et al*., 2014; Köster *et al*., 2025). While BIK1‐mediated activation of RBOHD is essential for initiating ROS signal initiation during PTI, the combined functions of CPK5 and CIPK26 are indispensable for propagating the ROS signal and mounting systemic immunity. Mutual phosphorylation of CIPK26 and CPK5 dramatically enhances their activity toward RBOHD, imposing a specific phospho‐code in RBOHD, which simultaneously sensitizes the NADPH oxidase to low Ca^2+^ levels and potentiates ROS generation, crucial for systemic immunity (Köster *et al*., 2025). These insights identify promising targets for synthetically improving crop pathogen resistance through precision editing.

In this context, a map‐based cloning approach identified *ZmWAKL*, encoding a cell‐wall‐associated receptor‐like kinase, as the causal gene at a main quantitative disease resistance locus against gray leaf spot (GLS), a major foliar disease of maize (Zhong *et al*., 2024). Subsequent molecular studies revealed that the ZmWAKL^Y^/ZmWIK receptor complex interacts with and phosphorylates the receptor‐like cytoplasmic kinase (RLCK) ZmBLK1, which in turn phosphorylates the downstream NADPH oxidase ZmRBOH4 to confer pathogen resistance. The obvious functional conservation of receptor/signaling/RBOH‐executor modules between Arabidopsis and important crops provides a strong potential for successfully translating mechanistic findings from *Arabidopsis* into practical, agronomically relevant applications in crop protection.

Notably, in Arabidopsis, another CPK, CPK28, contributes to immune homeostasis by promoting degradation of BIK1 (Monaghan *et al*., 2015). Additionally, this kinase functions in the vegetative‐to‐reproductive stage transition and in plant cold tolerance (Matschi *et al*., 2015; Ding *et al*., 2022). Molecularly, the phosphorylation status of Ser318 in CPK28 determines its Ca^2+^ sensitivity and provides a mechanism to decouple the activity of this kinase in immune signaling from its role in reproductive growth (Bredow *et al*., 2021). This dual function and the Ca^2+^‐sensitivity regulation of CPK28 appear to be evolutionarily conserved in crops. In rice, OsCPK4 and OsCPK18 (the orthologs of Arabidopsis CPK28) similarly phosphorylate OsRLCK176 (the ortholog of Arabidopsis BIK1) to promote its degradation. Both CPKs also affect yield‐related traits by regulating the vegetative‐to‐reproductive stage transition (Wang *et al*., 2018). Loss of CPK function in *cpk4* and *cpk18* mutants rendered these plants more pathogen resistant but impaired their growth and yield, highlighting the common growth–defense trade‐off in plants that complicates efforts to improve stress tolerance (Table S1) (Xie *et al*., 2014; Li *et al*., 2022). The MAP kinase OsMPK5 phosphorylates OsCPK4 and OsCPK18 at their C‐terminal residues S512 and T505, respectively, and this phosphorylation modulates the Ca^2+^ sensitivity of their activation, similar to what is observed with *Arabidopsis* CPK28 (Li *et al*., 2022). Notably, ablating this MAPK phosphorylation motif in OsCPK4 and OsCPK18 through genome editing allowed uncoupling the growth–defense trade‐off inherent in wild‐type OsCPK4/OsCPK18 function and created plants simultaneously exhibiting enhanced immunity and enhanced growth/yield. This breakthrough finding demonstrates that precision editing of phosphorylation sites allows synthetically fine‐tuning protein activity and regulatory circuits in a so far unprecedented way and showcases the potential of knowledge‐driven modification of phosphorylation sites in Ca^2+^ signaling components for crop improvement.

Similar to AtCPK28 in Arabidopsis and OsCPK4/OsCPK18 in rice, ZmCPK39 negatively regulates plant immunity in maize. A recent study of this kinase revealed that exploiting natural CPK variation also holds promise for enhancing crop pathogen resistance and uncovered an alternative approach to achieving optimal CPK activity fine‐tuning for avoiding growth–defense trade‐offs. Map‐based cloning of a major quantitative trait locus (QTL) conferring resistance to multiple foliar diseases, including gray leaf spot (GLS), northern leaf blight (NLB), and southern leaf blight (SLB), revealed that natural variation in ZmCPK39 is a key determinant of this agronomically highly relevant trait (Zhu *et al*., 2024b). ZmCPK39 phosphorylates and mediates the degradation of the transcription factor ZmDi19, which promotes the expression of ZmPR10, a gene encoding an antimicrobial protein (Zhu *et al*., 2024b). In a resistant maize line, a 3‐SNP variation in a *cis*‐regulatory element (CRE) of the ZmCPK39 promoter (ZmCPK39^Y32^) resulted in significantly (30–50%) reduced pathogen‐induced expression of ZmCPK39 compared with its expression in a susceptible line. This led to decreased phosphorylation and degradation of ZmDi19, thereby increasing ZmDi19 abundance. Importantly, pathogen‐resistant plants carrying the ZmCPK39^Y32^ allele exhibited no detectable negative effects on agronomic traits under uninfected conditions. This finding is notable since the complete loss of ZmCPK39 function causes dramatic developmental phenotypes, including severe dwarfism (Zhu *et al*., 2024a). Thus, the ZmCPK39^Y32^ allele appears to allow for optimal fine‐tuning of the growth–defense tradeoff, improving plant survival during fungal infections without compromising growth. This discovery suggests that targeted promoter editing to modulate ZmCPK39 expression could be a viable strategy for crop improvement, either as an alternative or complement to editing its phosphorylation sites. Most importantly, this study on maize, as well as the aforementioned study on rice CPKs, provides compelling proof‐of‐concept evidence that fine‐tuning the activity of Ca^2+^ signaling components at the level of gene expression or protein activity is a viable strategy for enhancing crop stress tolerance without reducing growth and yield. Such strategies overcome the fundamental limitations of previous biotechnological approaches, in which gene knockout or overexpression for enhancing stress tolerance often caused drastic growth and yield penalties.

In the second layer of plant defense, nucleotide‐binding domain leucine‐rich repeat proteins (NBS‐LRRs or NLRs) recognize pathogen effectors to trigger effector‐triggered immunity (ETI). Although PTI and ETI are initiated by distinct receptors, they mutually potentiate each other and share several overlapping signaling events, including the activation of Ca^2+^ signals (Ngou *et al*., 2021; Pruitt *et al*., 2021; Yuan *et al*., 2021). For instance, CPK5, which activates RBOHD in PTI, also functions in ETI (and systemic acquired immunity) by phosphorylating multiple downstream targets, including CBP60g and CAMTA3, which regulate the transcription of downstream genes (Fig. 1b) (Guerra *et al*., 2020; Kim *et al*., 2020; Sun *et al*., 2020, 2022; Liu *et al*., 2024). This indicates that Ca^2+^ signaling represents a critical convergence point for both PTI and ETI (Kim *et al*., 2022). Therefore, ETI‐related Ca^2+^ signaling processes emerge as promising targets for strategies aimed at enhancing crop immunity.

Most excitingly, recent research has revealed that NLRs, which function as effector receptors, ultimately oligomerize to form Ca^2+^‐permeable pores in the plasma membrane (Bi *et al*., 2021; Kim *et al*., 2022; Lee & Romeis, 2022; Parker *et al*., 2022; Huang *et al*., 2023). In *Arabidopsis*, recognition of a pathogen effector by the NLR HOPZ‐ACTIVATED RESISTANCE 1 (ZAR1) induces the formation of a large hetero‐oligomeric protein complex, known as the ZAR1 resistosome, which functions as a Ca^2+^‐permeable pore required for immunity (Fig. 1b) (Bi *et al*., 2021). The formation and function of resistosomes are conserved in crops. For example, the wheat CNL Sr35 (stem rust resistance gene 35) resistosome and the Arabidopsis ZAR1 resistosome are conserved in structure and function as Ca^2+^ permeable pores (Förderer *et al*., 2022). Despite considerable differences in the details of processing and function of individual CNLs, the emerging paradigm implicates that, in general, all NLR resistosomes converge on Ca^2+^ influxes into cells (Kim *et al*., 2022; Parker *et al*., 2022; Huang *et al*., 2023). Due to the novelty of this game‐changing discovery, many details regarding the regulation and genetic variation of NLRs await further elucidation. However, it is safe to predict that these Ca^2+^ signaling components of ETI hold great promise as targets for improving crops.

Finally, a most recent breakthrough study demonstrates that manipulating Ca^2+^ signaling in crops at the level of Ca^2+^ signal formation can not only help plants repel pathogens but also enhance their beneficial relationships with arbuscular mycorrhizal fungi and nitrogen‐fixing bacteria. The initiation and manifestation of these root endosymbioses generally involve the formation of nuclear Ca^2+^ oscillations. In the model legume *Medicago truncatula*, Ca^2+^ signal formation critically depends on the function of three paralogous MtCNGC15 channels, which form a complex with the regulatory protein MtDMI1 (Charpentier *et al*., 2016). By pursuing a TILLING‐based mutant screen approach combined with *in planta* Ca^2+^ imaging in *M. truncatula*, Cook and colleagues identified *cngc15* mutants (designated a *cngc* ^*15GOF*^) harboring a dominant mutation in the S1 helix of this channel (Cook *et al*., 2025). This mutation causes autoactivation of the MtCNGC15 Ca^2+^ channel and suffices to generate spontaneous low‐frequency Ca^2+^ oscillations in the absence of (legume‐specific) MtDMI1, leading to increased plant nutrient acquisition by enhancing arbuscular mycorrhiza (AM) and rhizobial colonization in *M. truncatula*. This MtDMI1‐independent activation of MtCNGC15 paved the way for transferring this auto‐activated AM‐enhancing mechanism into nonlegume crops. To achieve this, the authors again employed a TILLING approach to isolate dominant, autoactive *cngc15* mutants from the wheat species *Triticum aestivum* (*Tacngc15* ^*GOF*^) and *Triticum turgidum* (*Ttcngc15a* ^*GOF*^), and subsequently characterized these mutants under field conditions. Remarkably, both wheat *cngc15* ^*gov*^ lines exhibited enhanced AM colonization and increased nutrient acquisition even under field conditions with substantial inorganic fertilizer application. This finding is particularly noteworthy, as conventional fertilization practices typically suppress AM interactions in crops. Therefore, this breakthrough approach not only offers a novel strategy to enhance crop yield and productivity but also holds significant potential to support sustainable agricultural practices.

## III. A conceptual roadmap for harnessing the potential of Ca^2+^ signaling for crop improvement

The impact of abiotic and biotic stresses each year significantly reduces the theoretically possible crop yield – yields that could be achieved if crops were grown under optimal conditions (Fig. S1) (Bray *et al*., 2000). Therefore, enhancing crop stress resilience presents a tremendous opportunity to increase agricultural productivity. Before delving into potential future avenues for crop Ca^2+^ research, it is essential to consider why deepening our understanding and utilization of existing natural genetic variation in Ca^2+^ signaling components, combined with emerging opportunities for creating synthetic genetic variation, could provide unique and powerful strategies to optimize crop stress resilience and yield improvements.

### 1. Why is Ca^2+^ signaling uniquely poised to optimize agronomically relevant crop traits?

The first answer to this question lies in the genetic and physiological ‘architecture’ of modern crops. The onset of agricultural cultivation and domestication altered the strength and composition of selective forces that were previously exerted on wild plant species. For example, field cultivation practices likely reduced the selective pressure for competition with other species for light and nutrients. Simultaneously, human‐driven artificial selection aimed at increasing yield (e.g. larger seeds and fruits) and improving traits relevant to agricultural practices (e.g. reduced seed shattering) became prevalent. However, resilience to abiotic and biotic stressors is often less immediately obvious in the field and much more challenging to select for, even using modern breeding techniques. Therefore, active domestication selection over the past millennia and modern breeding over the past decades could only address the optimization of crop stress resilience to a limited extent. Although prolonged domestication and modern breeding have enhanced productivity‐boosting traits, they have also involved genetic bottlenecks that reduce genetic diversity, including the available allele pool that could confer resilience to adverse environmental and growth conditions (Zhang & Batley, 2020). Furthermore, epistatic relationships, combined with the focus on optimizing yield, may have further eroded alleles and genes that could provide stress resilience. For instance, in tomatoes, selection for larger fruit size in commercial cultivars favored an SOS2 allele that reduces salt tolerance, while an alternative salt‐tolerant SOS2 allele is still frequently found in wild tomatoes (Hong *et al*., 2023). Such limitations are not confined to stress resilience – negative epistasis between traits determining productivity has also been observed. For example, early selection for a MADS box gene allele that increases fruit size prevented the inbreeding of a favorable allele of another MADS box gene, which would reduce fruit drop, as this allele combination caused undesirable branching and sterility (Soyk *et al*., 2017).

The second answer to the question stems from the fundamental role and mechanisms of plant Ca^2+^ signaling. As an upstream regulatory hub, Ca^2+^ signaling has the unique capacity to modulate entire downstream response networks and to influence multiple physiological processes. Therefore, manipulating Ca^2+^ signaling can affect one specific downstream response, and could modulate multiple responses, or could even allow for the creation of new responses.

Principally, manipulating Ca^2+^ signaling could be achieved at two levels. (i) Manipulating Ca^2+^ signal generation: This involves controlling where, when, and how strongly and durably Ca^2+^ signals are generated by targeting Ca^2+^ channels. Strategies could include altering channel expression patterns, levels, regulatory mechanisms, or conductive efficiency. (ii) Optimizing Ca^2+^ signal decoding: This could be achieved by modifying the expression pattern or levels of Ca^2+^ sensors, their stability or activation status, or by altering their Ca^2+^ binding properties.

The elegance and promise of engineering Ca^2+^ signal decoding components stem from the inherent stimulus‐specificity and transient nature of Ca^2+^ signal formation. Normally, constitutive activation of a stress response pathway in crops enhances stress resilience but inevitably leads to severe growth retardation and reduced yield. However, in the case of a Ca^2+^‐regulated kinase, creating and expressing a kinase version with constitutively enhanced ‘biochemical activity’ (through mutations in regulatory phosphorylation sites or residues affecting kinase activity) would not necessarily result in a constitutively ‘biologically active’ kinase *in planta* without the corresponding stress stimulus. For example, a CIPK, which only interacts with its cognate CBL partner in a Ca^2+^‐dependent manner, would still require the formation of a transient Ca^2+^ signal induced by naturally occurring stress to trigger interaction with the CBL. This interaction then would confer (dependent on its identity) either plasma membrane or tonoplast targeting of the resulting CBL/CIPK complex for efficient target protein phosphorylation. An example of this principle is illustrated by the OsCIPK7 TILLING allele, which enhanced its kinase activity by affecting the activation‐loop folding of CIPK7, conferring enhanced chilling tolerance in rice plants without noticeable growth or yield penalties (Zhang *et al*., 2019). Similarly, in the case of a Ca^2+^‐dependent CPK/CDPK, even if the kinase is ‘biochemically hyperactive’, Ca^2+^ binding to the EF‐hand domains of its regulatory domain remains essential to trigger an intramolecular conformational change that releases the inhibitory blockade of its catalytic center by the pseudo‐substrate domain required for ‘biological activation’ (Liese & Romeis, 2013; Yip Delormel & Boudsocq, 2019). Consistent with this, overexpressing multiple CPKs has been shown to produce drought stress‐resistant plants without compromising growth (Schulz *et al*., 2021).

In addition to modulating enzyme activity, manipulating the Ca^2+^ binding affinity or the Ca^2+^ association/dissociation rates of either CBLs or CPKs could either enhance or reduce the threshold of Ca^2+^ concentration required for activation, effectively sensitizing or desensitizing stress responses, or could control the duration of Ca^2+^ decoder activation during a stress response (Lederer *et al*., 2025). As discussed in detail above, a proof‐of‐concept for the feasibility of such synthetic response pathway modification was already provided in the case of OsCPK18 and OsCPK4, where disrupting a specific C‐terminal S/T‐phosphorylation motif reduced their Ca^2+^‐sensitivity, allowing one to simultaneously improve rice yield and immunity (Li *et al*., 2022).

In essence, the stimulus specificity and transience of Ca^2+^ signal formation combined with the distinctive regulatory mechanisms of Ca^2+^ signal decoders provide the realistic perspective that a qualitatively novel class of improved crops – ‘smart crops’ – can be generated through knowledge‐based, precise modulation of Ca^2+^‐decoder activity, sensitivity, or specificity. Such smart crops would autonomously mount an optimized and stimulus‐specific stress response only when exposed to adverse abiotic or biotic environmental cues. With this capability, smart crops can preserve normal growth and resource allocation under optimal conditions while unlocking adaptive plasticity under stress to achieve an optimal balance of growth and defense/resilience. Harnessing the application potential of Ca^2+^ signaling in this way is a promising path for the intelligent breeding of such smart crops, which has recently been proposed as a core strategy and central goal for the future Breeding 5.0 generation of crops (Yu *et al*., 2024).

### 2. Experimental strategies for leveraging Ca^2+^ signaling for crop improvement

**Fig. 3:**
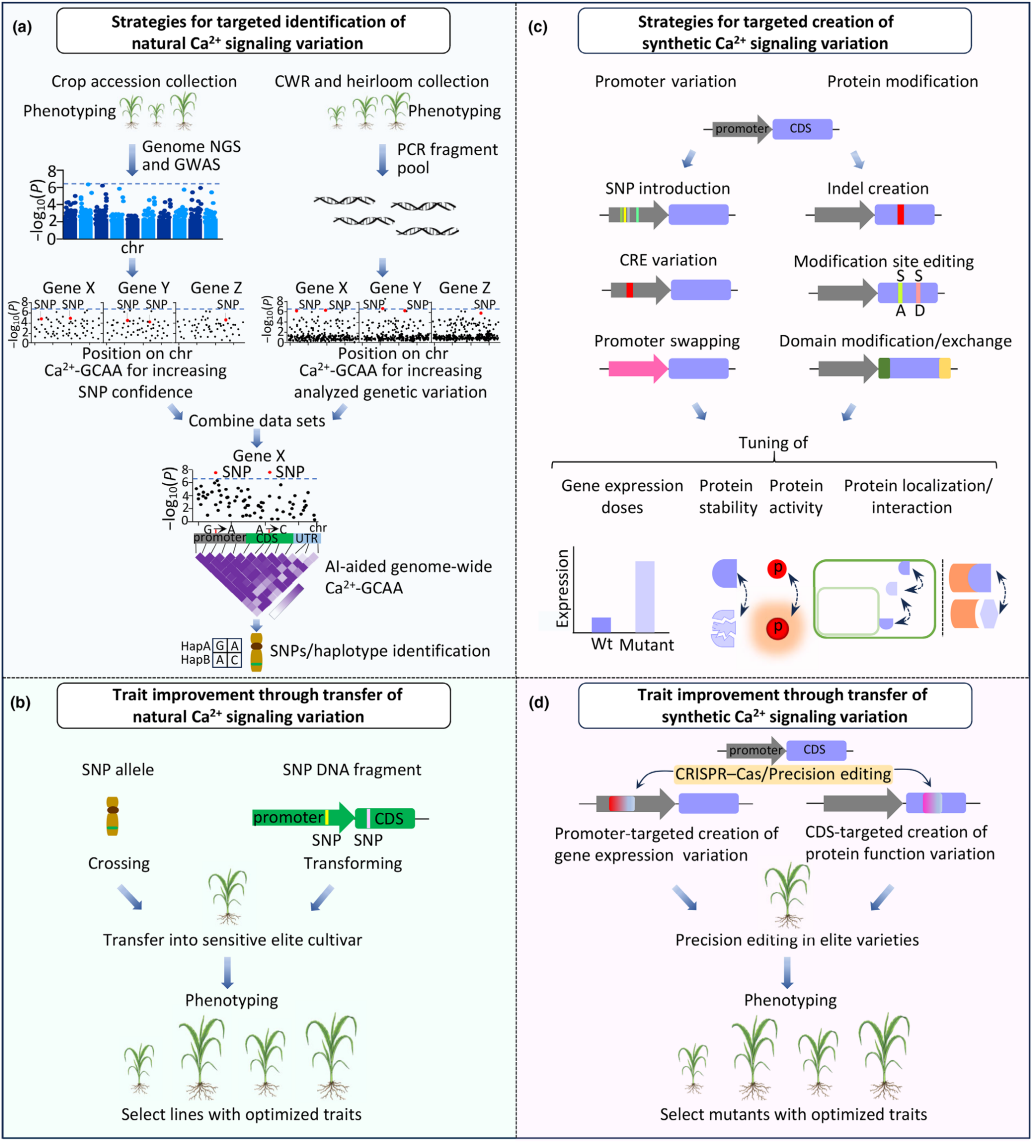
Strategies for identifying natural variation and creating synthetic variation in Ca^2+^ signaling to improve crop traits. (a) Utilizing crop accessions, crop wild relatives (CWR), and heirloom varieties, followed by phenotyping to assess traits. Genomic analysis involves next‐generation sequencing (NGS) combined with genome‐wide association studies (GWAS), and PCR‐fragment pools subjected to subsequent NGS and Ca^2+^‐GCAA (Ca^2+^ signaling gene‐centered association analysis), to identify genetic variations (e.g. SNPs, haplotypes) linked to Ca^2+^ signaling. AI‐aided genome‐wide Ca^2+^‐GCAA enhances the detection of significant SNPs and haplotypes (e.g. HapA, HapB). (b) Favorable SNPs are transferred to sensitive elite cultivars through crossing or transformation, followed by phenotyping to select lines with optimized traits, enabling trait improvement via natural Ca^2+^ signaling variation. (c) Deliberate creation of specific SNPs, modification of *cis*‐regulatory element (CRE) variations, and swapping of promoter sequences to modulate gene expression; or creation of indels, editing of modification sites, and modification/exchange of domains in coding sequences (CDS) to alter protein stability, activity, localization, or interaction. (d) Synthetic variations in promoters and CDS are generated via precision editing (e.g. CRISPR‐Cas) in elite varieties, followed by phenotyping to select mutants with optimized traits.

Which experimental approaches could be pursued to fully capitalize on the potential of Ca^2+^ signaling for crop improvement? In principle, two conceptually complementary, general strategies, which both would focus on the Ca^2+^ signaling gene space of crops, hold great promise of success. The first strategy aims at more efficiently exploring and identifying existing natural genetic variation with beneficial impacts on stress tolerance. The second approach focuses on synthetically creating genetic variation that enhances crop stress resilience, but is not present in the natural gene pool of crops (Fig. 3). Importantly, these approaches are not mutually exclusive and offer opportunities for synergism; for example, a beneficial natural allele identified in one crop species could be synthetically introduced into another species where it does not naturally exist.

Genome‐wide association studies (GWAS) and QTL mapping approaches exploring the existing natural variation of crops have already allowed, to some extent, to successfully harness the potential of Ca^2+^ signaling for identifying alleles, which positively impact plant stress‐related traits (Fig. 2; Table S1) (Ma *et al*., 2015; Zhu *et al*., 2024b). However, as noted, alleles conferring favorable stress‐related traits are often present at low frequencies in modern crop accessions. Additionally, the phenotypic and genetic robustness inherent in the network‐like character of Ca^2+^ signal decoding systems often causes a rather moderate manifestation of mutant and allele phenotypes, complicating their detection in large‐scale GWAS analyses. This phenomenon is further exacerbated by the existence of large gene families, such as CPKs, and their inherent ability to functionally compensate for genetic alterations in individual family members.

Consequently, in standard GWAS, potentially stress‐relevant SNPs may not be identified because they fail to meet the statistical significance thresholds to be recognized as being significant. However, ‘candidate gene association analyses’ (CGAA), which focus on specifically selected genes, can enhance detection power by incorporating additional weighting parameters. This approach has already successfully identified trait‐relevant SNPs in candidate genes that were not recognized in previous GWAS (Hu *et al*., 2024; Wang *et al*., 2024). While GWAS is an untargeted, hypothesis‐free, genome‐wide screening method, CGAA represents a rational approach where the targeted genes are selected based on *a priori* hypotheses about their function (David, 2021). Such hypotheses can result from previously characterized loss‐of‐function alleles but could, for example, also consider informative gene expression patterns or protein interaction data. Rather than performing CGAA focusing on a single gene, we propose to advance such studies toward a more comprehensive but still specific association analyses that focus on the complete gene space encoding Ca^2+^ signaling components in a crop genome: ‘Ca^2+^ signaling–candidate gene association analysis’ (Ca^2+^‐CGAA). Such Ca^2+^‐CGAA would allow for efficient and comprehensive in‐depth exploration of trait‐relevant genetic variation in any gene encoding a Ca^2+^ signaling component within a crop genome (Fig. 3a).

In addition to overcoming the limitations posed by rare allele frequencies of certain alleles, this approach should be complemented by an expanded Ca^2+^‐CGAA strategy that incorporates a broader genetic pool, particularly from CWRs and heirloom accessions. To this end, the genomic inventory of Ca^2+^ signaling genes from these CWR/heirloom collections would be specifically amplified via PCR. Next‐generation sequencing (NGS) of these candidate gene PCR‐fragment pools would then provide high‐quality, high‐coverage sequence data, enabling sensitive SNP detection (Fig. 3a). Given that the relatively compact Arabidopsis genome encodes over 100 Ca^2+^‐permeable channels and transporters, along with *c*. 250 EF‐hand Ca^2+^‐binding proteins, a similar approach in crops would involve the amplification and analysis of several hundred genes and their regulatory regions (Day *et al*., 2002; Edel & Kudla, 2015). Finally, the data from GWAS will be integrated with the data obtained from the CWR/heirloom collection, and jointly analyzed using a comprehensive artificial intelligence (AI)‐aided Ca^2+^‐CGAA approach. The development of iteratively trained predictive AI tools for Ca^2+^‐CGAA will significantly enhance the detection and predictive power of such data mining efforts. These predictive AI‐based large language models (LLMs) would function similarly to the recently developed protein language models, which process large datasets to learn the implicit ‘grammar’ of protein evolution (Ruffolo *et al*., 2025). To decode the complex statistical relationships of the occurrence and frequency of DNA sequence variations and trait‐related gene functions, these ‘allele impact language models’ would be iteratively trained with accumulating data on gene functions, drawn from studies in multiple species. These data would include, for example, insights about gene function obtained from mutant analyses, gene expression patterns, evolutionary trajectories, protein domain and *cis*‐regulatory element (CRE) conservation, SNP validation, and phenotypic penetrance. Iterative reasoning steps could, for instance, prioritize SNPs that occur more frequently in CWRs than in crop accessions, emphasizing genetic variation with potentially greater relevance for stress tolerance. In this way, AI‐aided Ca^2+^‐CGAA does not solely rely on statistical significance to identify trait‐relevant genetic variation, but also enhances the analysis by considering biological relevance. Moreover, the superior pattern recognition abilities of LLMs may allow for the identification of co‐occurring haplotype combinations across distinct genes, allowing for the detection of potential co‐selection and better understanding (and utilization) of the network‐like properties of Ca^2+^ signaling systems. Such AI‐based applications are already a reality in practical use. For example, the recently developed biological foundation model EVO2 enables accurate prediction of the functional impacts of genetic variation without the requirement for specific task‐specific finetuning (Brixi *et al*., 2025). Finally, experimentally validated favorable SNPs (or SNP combinations) can be introduced into stress‐sensitive elite cultivars via conventional breeding programs (through crossing) or genome editing techniques (by transformation) (Fig. 3b).

Novel genome editing tools, such as precision editing and HDR‐mediated editing, offer highly precise and efficient methodologies for fully exploiting natural genetic diversity, as well as for the knowledge‐based creation of synthetic genetic diversity for crop improvement. These approaches can target either the promoter regions of genes to modulate gene expression, or their coding sequences (CDS) to directly manipulate protein function (Fig. 3c).

Technically, it is now feasible to modulate gene expression by creating specific SNPs, modifying or introducing *cis*‐regulatory elements (CREs), or swapping complete promoter sequences. A recent study provided an impressive example of the power of such smart promoter editing. The study reported improved prime‐editing tools and their application in developing a Climate‐Responsive Optimization of Carbon Partitioning to Sinks (CROCS) strategy. This strategy involves the rational manipulation of cell‐wall invertase (CWIN) expression in fruit and cereal crops (Lou *et al*., 2025). Using their high‐efficiency prime‐editing tools, the authors precisely knocked in a 10‐bp heat‐shock element (HSE) into the promoters of cell‐wall invertase genes (CWINs) in elite rice and tomato cultivars. The HSE insertion resulted in heat‐responsive upregulation in both controlled and field environments, enhancing carbon partitioning to grain and fruit. This resulted in per‐plot yield increases of 25% in rice and 33% in tomatoes compared with heat‐stressed controls without compromising fruit quality.

In the CDS, regulatory domains can be deleted, exchanged, or added, and specific regulatory amino acids can be altered through base pair editing (Fig. 3c). Such edits allow fine‐tuning of protein stability, enzyme activity, protein localization, and protein–protein interactions, all of which can enhance stress resilience without causing yield penalties. Excitingly, precision editing and HDR editing enable the synthetic creation of novel amino acid residues that naturally do not occur in proteins, offering the potential to create elite alleles that go beyond natural evolution. Notably, novel AI‐enabled approaches for protein design hold great promise for further enhancing the power of creating synthetic variation by enabling access to areas of the crop fitness landscape that have remained unexplored during natural evolution (Li *et al*., 2025). Specifically, AI‐aided protein design, AI‐based prediction of protein function, and AI‐aided impact prediction of mutating protein‐phosphorylation sites could greatly accelerate progress in improving crop traits (Li *et al*., 2025; Notin, 2025). Because of the precision of novel genome‐editing technologies, such manipulations can be performed directly in existing elite varieties. Alternatively, if such genotypes are recalcitrant to transformation, they can be created in transformable genotypes and included in breeding programs (Fig. 3d). Collectively, by leveraging both the naturally evolved and the synthetically engineered genetic diversity of crops, ‘applied Ca^2+^ signaling research’ can substantially expedite next‐generation breeding approaches. Importantly, optimizing Ca^2+^ signaling activity through newly identified natural or synthetically created novel alleles enables the development of crops with enhanced stress tolerance without the yield trait‐offs that have previously hindered crop improvement through mutant or overexpression approaches.

## IV. Current challenges and future perspectives for Ca^2+^ signaling research in crops

Intensive research, particularly in model organisms, has over the past 30 years transformed our understanding of plant Ca^2+^ signaling from being regarded as an ‘interesting phenomenon’ to being appreciated as a central feature in almost all aspects of plant biology. Most importantly, it is now recognized as a crucial determinant of plant resilience to both abiotic and biotic stresses. In the past decade, expanding this research to major crop species has provided strong evidence for the functional conservation of key Ca^2+^ signaling components and circuits, establishing their practical relevance for crop breeding and the improvement of agronomically significant traits. This knowledge, combined with breakthroughs in relevant biotechnological methods, has advanced the field to a point where the focus can increasingly shift from ‘understanding Ca^2+^ signaling’ in crop biology to ‘applying Ca^2+^ signaling’ for crop trait enhancement and for creating smart crops. However, despite these exciting prospects, several challenges remain that must be addressed to fully unlock the transformative potential of Ca^2+^ signaling research.

One major challenge is the limited functional characterization of the gene space encoding Ca^2+^ signaling components. In model species like Arabidopsis, this gene space encompasses *c*. 100 Ca^2+^‐permeable channels and transporters, and *c*. 250 EF‐hand‐containing Ca^2+^‐binding proteins. Crop genomes, however, tend to be much more complex, often with expanded gene families (Day *et al*., 2002; Edel *et al*., 2017). Furthermore, even in Arabidopsis, research has mainly focused on a few prominent gene families such as CPKs, CIPKs, and CaMs. Nevertheless, even these gene families remain incompletely characterized to date (T. Wang *et al*., 2023). However, the rapid development of highly efficient biotechnological technologies now provides powerful tools to comprehensively explore the biological functions represented in this gene space. For example, advances in CRISPR library construction, including barcode‐tagged and multi‐targeted libraries, offer more streamlined and efficient approaches to creating and screening mutant libraries in crop species, allowing for a comprehensive understanding of the contribution of Ca^2+^ signaling genes to crop stress tolerance (Chen *et al*., 2022; Berman *et al*., 2025).

In Arabidopsis, the use of biosensors to characterize Ca^2+^ signals in response to environmental cues, as well as to study the impact of different mutants on Ca^2+^ signal formation, has advanced considerably. Such studies have historically, and still do today, often used the luminescent Ca^2+^ reporter aequorin (Kiegle *et al*., 2000; Moore *et al*., 2002; Zhao *et al*., 2023; Sun *et al*., 2025; S. Zhang *et al*., 2025). However, due to their superior sensitivity and spatial resolution, such research increasingly involves the use of fluorescent Ca^2+^ reporter proteins, such as the ratiometric YC3.6 Ca^2+^ indicator or the intensiometric R‐GECO and GCaMP indicators (Krebs *et al*., 2012; Waadt *et al*., 2017). Recent developments in dual‐reporting Ca^2+^ biosensors have enabled the ratiometric normalization of R‐GECO or GCaMP measurements. This enhances the reliability of these measurements and enables the simultaneous investigation of cellular dynamics of Ca^2+^ and of the stress hormone ABA (Waadt *et al*., 2017, 2020). Notably, mutant screens performed in genetic backgrounds expressing suitable Ca^2+^ reporter proteins, such as Aequorin, successfully identified mutants that affect Ca^2+^ signal formation, and screens using advanced fluorescent Ca^2+^ reporters, such as YC3.6, enabled the direct combination of macroscopic phenotypic analysis and Ca^2+^ signal phenotype analysis (Yuan *et al*., 2014; Wu *et al*., 2020; Cook *et al*., 2025). However, to date, relatively few reports have been published on the application of Ca^2+^ biosensors in crops. Examples of such studies include the use of aequorin, YC3.6, and different versions of GCaMPs in crops such as tomatoes, barley, and rice (Behera *et al*., 2015; Ma *et al*., 2015; Zhang *et al*., 2015; Li *et al*., 2017; J. Wang *et al*., 2021; Giridhar *et al*., 2022; H. Wang *et al*., 2023; Harada *et al*., 2025). This relative paucity of reports characterizing Ca^2+^ responses in crops is likely due to the challenges associated with the need for imaging systems that allow the observation of larger samples. For example, the roots of maize and rice are much larger than the roots of Arabidopsis. Furthermore, the intricate structure of crop roots, which harbor multiple cortex layers and an exodermis, which is absent in Arabidopsis, complicates the detection of Ca^2+^ signals in inner root cell layers. Accordingly, we and others have been unable to observe Ca^2+^ signals in response to NaCl stress in primary roots of rice plants expressing the Ca^2+^ indicator YC3.6 (Li *et al*., 2017). Fortunately, recent advances in developing highly sensitive and reliable ultra‐sensitive Ca^2+^ reporter proteins that allow for simplified measurement technology now hold promise for significantly expanding such studies to a wider range of crop species and stress stimuli (Waadt *et al*., 2021; Hagihara *et al*., 2022; Aratani *et al*., 2023; Gu *et al*., 2025; Hao *et al*., 2025). Additionally, further advances in microscopic live‐cell imaging techniques for complex tissues will facilitate the study of Ca^2+^ dynamics in crops. Considering the recent progress in long‐distance signaling in Arabidopsis, translating this to crops will require tailored microscopic and macroscopic solutions and necessitate collaboration between plant biologists and optical engineers. Moreover, substantial improvements in spatio‐temporal resolution for characterizing the expression and function of Ca^2+^ signaling components are still needed. The rapid advancement of single‐cell sequencing and other spatial‐omics technologies, along with significant advances in biosensor development for protein activity and second messenger/metabolite detection, offers an exciting opportunity to improve our understanding of Ca^2+^ signaling and its relevance for stress resilience (Bischof *et al*., 2017; W. Li *et al*., 2019; Liese *et al*., 2024; Zhu *et al*., 2025).

Another significant challenge is the limited biochemical knowledge of plant proteins, particularly regarding their Ca^2+^ binding properties (e.g. affinity and dissociation constants) and enzymatic activities (e.g. kinase activity). Classical protein‐biochemical methods to obtain such quantitative data are expensive, time‐consuming, and tedious, and despite their fundamental importance, such investigations are not always sufficiently supported by funding agencies and appropriately appreciated by journals. Technological advances, such as microscale thermophoresis for Ca^2+^ binding characterization and enzyme‐activation biosensors, present a viable path to overcome this bottleneck of essential information (Hochmal *et al*., 2016; Sparks & Fratti, 2019; Liese *et al*., 2024). Additionally, the lack of structural knowledge for plant proteins, particularly Ca^2+^‐regulated proteins, remains a challenge. Even with advanced structural prediction programs like AlphaFold2, the dynamic behavior of flexible domains such as EF‐hands is difficult to model accurately without closely related experimentally determined reference structures. Focusing on highly relevant target proteins to provide suitable reference structures, combined with AI‐based machine learning approaches for predicting protein function and structural dynamics, may help improve the predictive abilities of these programs.

In summary, despite significant progress in Ca^2+^ signaling research, several challenges currently hamper its widespread application in crop breeding. Our understanding of Ca^2+^ signaling networks in major crops, including key agricultural species like soybean, remains fragmented and incomplete. Identifying the function of the full complement of Ca^2+^ signaling components, elucidating their regulatory interactions, and understanding how genetic variation impacts network function are critical knowledge gaps that must be addressed. Moreover, although translating discoveries from model plants like Arabidopsis to crop species appears feasible, species‐specific differences in signaling mechanisms and gene regulation mean that a full understanding of the functional repertoire of Ca^2+^ signaling in crops cannot be achieved by simply extrapolating from model plants. Therefore, a focused and prioritized research effort in suitable model crop species, such as soybean for dicots and rice for monocots, is essential to overcome these limitations. Despite these challenges, Ca^2+^ signaling holds immense potential for revolutionizing crop improvement, offering multiple strategies to enhance stress resilience and yield stability. By identifying key regulatory nodes, deciphering the molecular basis of natural variations, and refining gene editing strategies, we can develop innovative solutions to safeguard global food security in the face of increasing environmental challenges.

## Competing interests

None declared.

## Disclaimer

The New Phytologist Foundation remains neutral with regard to jurisdictional claims in maps and in any institutional affiliations.

## Supporting information


**Fig. S1** The impact of abiotic and biotic stress on crop yield.
**Table S1** Experimentally characterized potential target loci for developing resilient crops.
Please note: Wiley is not responsible for the content or functionality of any Supporting Information supplied by the authors. Any queries (other than missing material) should be directed to the *New Phytologist* Central Office.
