# Supplementary material for: Calcium signaling in crops
Source: New Phytol. 2025 Dec 9;249(4):1644–58. doi: 10.1111/nph.70796 (PMC12824602; doi:10.1111/nph.70796)
Supplement: Supplementary file 1 — Fig. S1 The impact of abiotic and biotic stress on crop yield. Table S1 Experimentally characterized potential target loci for developing resilient crops. Please note: Wiley is not responsible for the content or functionality of any Supporting Information supplied by the authors. Any queries (other than missing material) should be directed to the New Phytologist Central Office. [file NPH-249-1644-s001.pdf]

***New Phytologist* Supporting Information**

Article title: Calcium signaling in crops

Authors: Chunxia Zhang, Yang Song and Jörg Kudla

Article acceptance date: 28 October 2025

The following Supporting Information is available for this article:

Fig. S1 The impact of abiotic and biotic stress on crop yield.

Yield loss of the main global economic crops due to abiotic stress and biotic stress. Abiotic stress including but not limited to salt, drought, flooding and extreme temperatures. Biotic stress including weeds, insects and diseases (Redrawn from Bray et al., 2000).

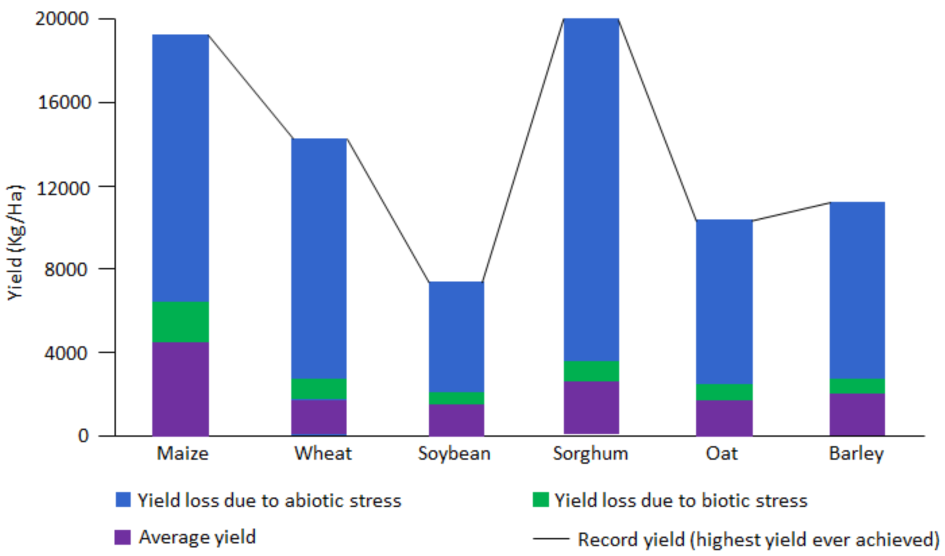

Table S1 Experimentally characterized potential target loci for creating stress resilient crops. This table provides detailed information and references for loci indicated in Fig. 2 in the main text.

Crop	Species	Trait	Ca^{2+} signaling component	Targets	Function	Reference
Rice	*Oryza sativa*	Salt	OsCPK5/ OsCPK13	OsMPK3/6	Positive regulator to salt stress	Su *et al*. (2024)
	Oryza sativa	Oxidative	OsCPK12	OsCATA and OsCATC	Overexpression of OsCPK12 enhance the tolerance to oxidative stress	Wang *et al*. (2024)
	Oryza sativa	Heat/Cold	OsCNGC14 /OsCNGC16	-	Required for heat and chilling tolerance	Cui *et al*. (2020)
	Oryza sativa		OsCBL7/8-OsCIPK7	OsCRT3	Required for chilling tolerance	Guo *et al*. (2023)
	Oryza sativa		OsCNGC9	-	Overexpression enhanced cold tolerance	Wang *et al.* (2021)
	Nipponbare		OsCPK24	OsGrx10	Overexpression enhanced cold tolerance	Liu *et al*. (2018)
	Oryza sativa		OsCML16	OsPILS7a	Positively regulates primary root growth under chilling stress	Liu *et al*. (2024)
	Oryza sativa	Pathogen resistance	OsCNGC9	OsRLCK185	Regulates the resistance to rice blast disease	Wang *et al.* (2019)
	Oryza sativa		OsCPK17	OsPUB12-OsRLCK176	Maintaining rice immune homeostasis.	Mou *et al*. (2024)
	Oryza sativa		ROD1	CatB	Regulates ROS homeostasis to suppress plant immunity	Gao *et al*. (2021)
	Oryza sativa		OsCPK18/ OsCPK4	OsMPK5	Fine tunes the growth-defence trade off	Li *et al*. (2022a)
Maize	*Zea mays*	Salt	ZmCBL8	ZmSOS1	Mutants sensitive to salt stress	Zhou *et al*. 2022

	Zea mays	Saline-alkaline	ZmNSA1	-	Negatively regulates saline-alkaline tolerance	Cao *et al*. (2020)
	Zea mays	Drought	ZmCPK35/ ZmCPK37	ZmSLAC1	Overexpression lines increased yield under drought stress condition	Li *et al*. (2022c)
	Zea mays		ZmCCaMK	ZmBSK1	Enhance drought tolerance	Liu *et al*. (2021)
	Zea mays		ZmCPK2	ZmCPK17	A negative regulator of drought resistance	Hu *et al*. (2024)
	Zea mays		ZmCIPK33	ZmSnRK2.10	Overexpression of ZmCIPK33 enhance drought resistance	Jiang *et al*. (2025)
	Zea mays *Zea mays*	Heat	ZmCDPK7	sHSP17.4 and RBOHB	Overexpression of ZmCDPK7 enhance thermotolerance	Zhao *et al*. (2021)
	Zea mays	Cold	CPK17	COOL1	Negatively regulates cold tolerance by phosphorylation of COOL1	Zeng *et al*. (2025)
	Zea mays	Pathogen resistance	ZmCPK39	ZmDi19-ZmPR10	Decrease the abundance of ZmCPK39 enhancing maize resistance to foliar diseases	Zhu *et al*. (2024b)
	Zea mays	Plant height	ZmCPK39	ZmKnox2	ZmCPK39-knockout mutants reducing plant height	Zhu *et al*. (2024a)
Tomato	*Solanum lycopersicum*	Salt	SlSOS2	SlABI4	Natural variation in the promoter region of SlSOS2 was associated with root Na^+/K^+ ratio and the loss of salt resistance	Hong *et al*. (2023)
	Solanum lycopersicum		SlCIPK23-SlCBL1/9	SlSKOR	Negatively regulate SlSKOR to affect root K^+ uptake and its translocation to the shoot	Nieves-Cordones *et al*. (2023)

	Solanum lycopersicum		SlCBL10	SlTPC1, SlAVP1 and SlVHA-A1	Mutation of SlCBL10 leads to the salt-hypersensitivity	Egea *et al*. (2018)
	Solanum lycopersicum	Heat	CPK28	APX2	Mutation of CPK28 impairs thermotolerance	Hu *et al*. (2021)
	Solanum lycopersicum	Cold	CaM6	ICE1	Silencing CaM6 enhancing cold tolerance.	Lin *et al*. (2023)
	Solanum lycopersicum	Pathogen resistance	CBL10-CIPK6	-	Overexpression of tomato CIPK6 in *N. benthamiana* leaves leading to ROS accumulation	de la Torre *et al*. (2013)
	Solanum		OSCA4.1	-	Negative regulator of resistance to PepMV	Ruiz-Ramon *et al*. (2023)
	Solanum lycopersicum	Sugar	SlCDPK27/ SlCDPK26	SlSUS3	Control fruit sugar content	Zhang *et al*. 2024
Wheat	*Triticum aestivum*	Pathogen resistance	TaCAMTA4	TaCAT1	Silencing of TaCAMTA4 enhanced *Puccinia triticina Erikss* resitance	Sun *et al*. (2024)
	Triticum aestivum		TaCIPK14	-	Knockdown of TaCIPK14 enhanced resistance to Pst	He *et al*. (2023)
	Triticum aestivum		TaCaM3	TaBln1	Silencing TaCaM3 reduced resistance to the Pst	Guo *et al*. (2022)
	Triticum aestivum		TaCIPK10	TaNH2	Overexpression of TaCIPK10 enhanced resistance to *Pst*	Liu *et al*. (2019)
	Triticum aestivum and Triticum turgidum	Symbiosis	TaCNGC15a and TtCNGC15a	-	Mutant increase in arbuscular mycorrhiza colonization	Cook *et al*. (2025)
Soybean	*Glycine soja*	Alkaline and salt stress	GsACA1	-	Overexpression GsACA1 enhanced tolerance to alkaline and salt stress	Sun *et al*. (2016)
	Glycine max	Pathogen resistance	GmCDPK38	-	Knock out GmCDPK38 causes delayed	Li *et al*. (2022b)

					flowering and enhanced resistance to the common cutworm in soybean	
	Glycine soja and Glycine max	Hard-seededness	GmHs1-1	-	Control hard-seededness in soybean	Sun *et al*. (2015)

References for Table S1

Bray E A, Bailey-Serres J and Weretilnyk E. 2000. Responses to abiotic stress. In: Gruissem, W. and Jones, R, eds. *Biochemistry & molecular biology of plants*. American Society of Plant Physiologists, Rockville, 1158-1203.

Cao Y, Zhang M, Liang X, Li F, Shi Y, Yang X, Jiang C. 2020. Natural variation of an EF-hand Ca^{2+}-binding-protein coding gene confers saline-alkaline tolerance in maize. *Nature Communications* **11**: 186.

Cook NM, Gobbato G, Jacott CN, Marchal C, Hsieh CY, Lam AHC, Simmonds J, Del Cerro P, Gomez PN, Rodney C, et al. 2025. Autoactive CNGC15 enhances root endosymbiosis in legume and wheat. *Nature* **638**, 752–759.

Cui Y, Lu S, Li Z, Cheng J, Hu P, Zhu T, Wang X, Jin M, Wang X, Li L, et al. 2020. CYCLIC NUCLEOTIDE-GATED ION CHANNELs 14 and 16 Promote Tolerance to Heat and Chilling in Rice. *Plant Physiology* **183**: 1794-1808.

de la Torre F, Gutierrez-Beltran E, Pareja-Jaime Y, Chakravarthy S, Martin GB, del Pozo O. 2013. The tomato calcium sensor Cbl10 and its interacting protein kinase Cipk6 define a signaling pathway in plant immunity. *Plant Cell* **25**: 2748-2764.

Egea I, Pineda B, Ortiz-Atienza A, Plasencia FA, Drevensek S, Garcia-Sogo B, Yuste-Lisbona FJ, Barrero-Gil J, Atares A, Flores FB, et al. 2018. The SlCBL10 Calcineurin B-Like Protein Ensures Plant Growth under Salt Stress by Regulating Na^{+} and Ca^{2+} Homeostasis. *Plant Physiology* **176**: 1676-1693.

Gao M, He Y, Yin X, Zhong X, Yan B, Wu Y, Chen J, Li X, Zhai K, Huang Y, et al. 2021. Ca^{2+} sensor-mediated ROS scavenging suppresses rice immunity and is exploited by a fungal effector. *Cell* **184**: 5391-5404.

Guo S, Zhang Y, Li M, Zeng P, Zhang Q, Li X, Xu Q, Li T, Wang X, Kang Z, et al. 2022. TaBln1, a member of the Blufensin family, negatively regulates wheat resistance to stripe rust by reducing Ca^{2+} influx. *Plant Physiology* **189**: 1380-1396.

Guo X, Zhang D, Wang Z, Xu S, Batistic O, Steinhorst L, Li H, Weng Y, Ren D, Kudla J, et al. 2023. Cold-induced calreticulin OsCRT3 conformational changes promote OsCIPK7 binding and temperature sensing in rice. *EMBO Journal* **42**: e110518.

He F, Wang C, Sun H, Tian S, Zhao G, Liu C, Wan C, Guo J, Huang X, Zhan G, et al. 2023. Simultaneous editing of three homoeologues of TaCIPK14 confers broad-spectrum resistance to stripe rust in wheat. *Plant Biotechnology Journal* **21**: 354-368.

Hong Y, Guan X, Wang X, Kong D, Yu S, Wang Z, Yu Y, Chao ZF, Liu X, Huang S, et al. 2023. Natural variation in SlSOS2 promoter hinders salt resistance during tomato domestication. *Horticulture Research* **10**: uhac244.

Hu X, Cheng J, Lu M, Fang T, Zhu Y, Li Z, Wang X, Wang Y, Guo Y, Yang S, et al. 2024. Ca^{2+}-independent ZmCPK2 is inhibited by Ca^{2+}-dependent ZmCPK17 during drought response in maize. *Journal of Integrative Plant Biology* **66**: 1313-1333.

Hu Z, Li J, Ding S, Cheng F, Li X, Jiang Y, Yu J, Foyer CH, Shi K. 2021. The protein kinase CPK28 phosphorylates ascorbate peroxidase and enhances thermotolerance in tomato. *Plant Physiology* **186**: 1302-1317.

Jiang S, Sun Z, Feng Z, Qi Y, Chen H, Wang Y, Qi J, Guo Y, Yang S, Gong Z. 2025. ZmCIPK33 and ZmSnRK2.10 mutually reinforce the abscisic acid signaling pathway for combating drought stress in maize. *Journal of Integrative Plant Biology* **67**: 1787-1804.

Li H, Zhang Y, Wu C, Bi J, Chen Y, Jiang C, Cui M, Chen Y, Hou X, Yuan M, et al. 2022a. Fine-tuning OsCPK18/OsCPK4 activity via genome editing of phosphorylation motif improves rice yield and immunity. *Plant Biotechnology Journal* **20**: 2258-2271.

Li X, Hu D, Cai L, Wang H, Liu X, Du H, Yang Z, Zhang H, Hu Z, Huang F, et al. 2022b. CALCIUM-

DEPENDENT PROTEIN KINASE38 regulates flowering time and common cutworm resistance in soybean. *Plant Physiology* **190**: 480-499.

Li XD, Gao YQ, Wu WH, Chen LM, Wang Y. 2022c. Two calcium-dependent protein kinases enhance maize drought tolerance by activating anion channel ZmSLAC1 in guard cells. *Plant Biotechnology Journal* **20**: 143-157.

Lin R, Song J, Tang M, Wang L, Yu J, Zhou Y. 2023. CALMODULIN6 negatively regulates cold tolerance by attenuating ICE1-dependent stress responses in tomato. *Plant Physiology* **193**: 2105-2121.

Liu L, Xiang Y, Yan J, Di P, Li J, Sun X, Han G, Ni L, Jiang M, Yuan J, et al. 2021. BRASSINOSTEROID-SIGNALING KINASE 1 phosphorylating CALCIUM/CALMODULIN-DEPENDENT PROTEIN KINASE functions in drought tolerance in maize. *New Phytologist* **231**: 695-712.

Liu P, Guo J, Zhang R, Zhao J, Liu C, Qi T, Duan Y, Kang Z, Guo J. 2019. TaCIPK10 interacts with and phosphorylates TaNH2 to activate wheat defense responses to stripe rust. *Plant Biotechnology Journal* **17**: 956-968.

Liu S, Zheng Y, Zhao L, Gulam M, Ullah A, Xie G. 2024. CALMODULIN-LIKE16 and PIN-LIKES7a cooperatively regulate rice seedling primary root elongation under chilling. *Plant Physiology* **195**: 1660-1680.

Liu Y, Xu C, Zhu Y, Zhang L, Chen T, Zhou F, Chen H, Lin Y. 2018. The calcium-dependent kinase OsCPK24 functions in cold stress responses in rice. *Journal of Integrative Plant Biology* **60**: 173-188.

Mou B, Zhao G, Wang J, Wang S, He F, Ning Y, Li D, Zheng X, Cui F, Xue F, et al. 2024. The OsCPK17-OsPUB12-OsRLCK176 module regulates immune homeostasis in rice. *Plant Cell* **36**: 987-1006.

Nieves-Cordones M, Amo J, Hurtado-Navarro L, Martinez-Martinez A, Martinez V, Rubio F. 2023. Inhibition of SlSKOR by SlCIPK23-SlCBL1/9 uncovers CIPK-CBL-target network rewiring in land plants. *New Phytologist* **238**: 2495-2511.

Ruiz-Ramon F, Rodriguez-Sepulveda P, Breto P, Donaire L, Hernando Y, Aranda MA. 2023. The tomato calcium-permeable channel 4.1 (SlOSCA4.1) is a susceptibility factor for pepino mosaic virus. *Plant Biotechnology Journal* **21**: 2140-2154.

Su S, Jiang Y, Zhu X, Yu S, Wang F, Xue L, Cui H. 2024. Calcium-dependent protein kinases 5 and 13 enhance salt tolerance in rice by directly activating OsMPK3/6 kinases. *Plant Physiology* **196**: 3033-3047.

Sun L, Miao Z, Cai C, Zhang D, Zhao M, Wu Y, Zhang X, Swarm SA, Zhou L, Zhang ZJ, et al. 2015. GmHs1-1, encoding a calcineurin-like protein, controls hard-seededness in soybean. *Nature Genetics* **47**: 939-943.

Sun M, Jia B, Cui N, Wen Y, Duanmu H, Yu Q, Xiao J, Sun X, Zhu Y. 2016. Functional characterization of a Glycine soja Ca^{2+} ATPase in salt-alkaline stress responses. *Plant Molecular Biology* **90**: 419-434.

Sun T, Ma N, Jiao Y, Wang Q, Wang Q, Liu N, Chen Y, Han S, Hou C, Wang R, et al. 2024. TaCAMTA4 negatively regulates H2O2-dependent wheat leaf rust resistance by activating catalase 1 expression. *Plant Physiology* **196**: 2078-2088.

Wang B, Xue P, Zhang Y, Zhan X, Wu W, Yu P, Chen D, Fu J, Hong Y, Shen X, et al. 2024. OsCPK12 phosphorylates OsCATA and OsCATC to regulate H_2O_2 homeostasis and improve oxidative stress tolerance in rice. *Plant Communication* **5**: 100780.

Wang J, Liu X, Zhang A, Ren Y, Wu F, Wang G, Xu Y, Lei C, Zhu S, Pan T, et al. 2019. A cyclic nucleotide-gated channel mediates cytoplasmic calcium elevation and disease resistance in rice. *Cell Research* **29**: 820-831.

Wang J, Ren Y, Liu X, Luo S, Zhang X, Liu X, Lin Q, Zhu S, Wan H, Yang Y, et al. 2021. Transcriptional activation and phosphorylation of OsCNGC9 confer enhanced chilling tolerance in rice. *Molecular Plant* **14**: 315-329.

Zeng R, Shi Y, Guo L, Fu D, Li M, Zhang X, Li Z, Zhuang J, Yang X, Zuo J, et al. 2025. A natural variant of COOL1 gene enhances cold tolerance for high-latitude adaptation in maize. *Cell* **188**: 1315-1329.

Zhang J, Lyu H, Chen J, Cao X, Du R, Ma L, Wang N, Zhu Z, Rao J, Wang J, et al. 2024. Releasing a sugar brake generates sweeter tomato without yield penalty. *Nature* **635**:647–656.

Zhao Y, Du H, Wang Y, Wang H, Yang S, Li C, Chen N, Yang H, Zhang Y, Zhu Y, et al. 2021. The calcium-dependent protein kinase ZmCDPK7 functions in heat-stress tolerance in maize. *Journal of Integrative Plant Biology* **63**: 510-527.

Zhou X, Li J, Wang Y, Liang X, Zhang M, Lu M, Guo Y, Qin F, Jiang C. 2022. The classical SOS pathway confers natural variation of salt tolerance in maize. *New Phytologist* **236**: 479-494.

Zhu M, Guo C, Zhang X, Liu Y, Jiang X, Chen L, Xu M. 2024a. The maize ZmCPK39-ZmKnox2 module regulates plant height. *aBIOTECH* **5**: 356-361.

Zhu M, Zhong T, Xu L, Guo C, Zhang X, Liu Y, Zhang Y, Li Y, Xie Z, Liu T, et al. 2024b. The ZmCPK39-ZmDi19-ZmPR10 immune module regulates quantitative resistance to multiple foliar diseases in maize. *Nature Genetics* **56**: 2815–2826.
